# High-density marker imputation accuracy in sixteen French cattle breeds

**DOI:** 10.1186/1297-9686-45-33

**Published:** 2013-09-03

**Authors:** Chris Hozé, Marie-Noëlle Fouilloux, Eric Venot, François Guillaume, Romain Dassonneville, Sébastien Fritz, Vincent Ducrocq, Florence Phocas, Didier Boichard, Pascal Croiseau

**Affiliations:** 1 INRA, UMR 1313 Génétique Animale et Biologie Intégrative, 78350 Jouy-en-Josas, France; 2 AgroParisTech, UMR1313 Génétique Animale et Biologie Intégrative, 75231 Paris 05, France; 3 Union Nationale des Coopératives agricoles d’Elevage et d’Insémination Animale, 149 rue de Bercy, 75595 Paris Cedex 12, France; 4 Institut de l’Elevage, 149 rue de Bercy, 75595 Paris Cedex 12, France

## Abstract

**Background:**

Genotyping with the medium-density Bovine SNP50 BeadChip^®^ (50K) is now standard in cattle. The high-density BovineHD BeadChip^®^, which contains 777 609 single nucleotide polymorphisms (SNPs), was developed in 2010. Increasing marker density increases the level of linkage disequilibrium between quantitative trait loci (QTL) and SNPs and the accuracy of QTL localization and genomic selection. However, re-genotyping all animals with the high-density chip is not economically feasible. An alternative strategy is to genotype part of the animals with the high-density chip and to impute high-density genotypes for animals already genotyped with the 50K chip. Thus, it is necessary to investigate the error rate when imputing from the 50K to the high-density chip.

**Methods:**

Five thousand one hundred and fifty three animals from 16 breeds (89 to 788 per breed) were genotyped with the high-density chip. Imputation error rates from the 50K to the high-density chip were computed for each breed with a validation set that included the 20% youngest animals. Marker genotypes were masked for animals in the validation population in order to mimic 50K genotypes. Imputation was carried out using the Beagle 3.3.0 software.

**Results:**

Mean allele imputation error rates ranged from 0.31% to 2.41% depending on the breed. In total, 1980 SNPs had high imputation error rates in several breeds, which is probably due to genome assembly errors, and we recommend to discard these in future studies. Differences in imputation accuracy between breeds were related to the high-density-genotyped sample size and to the genetic relationship between reference and validation populations, whereas differences in effective population size and level of linkage disequilibrium showed limited effects. Accordingly, imputation accuracy was higher in breeds with large populations and in dairy breeds than in beef breeds. More than 99% of the alleles were correctly imputed if more than 300 animals were genotyped at high-density. No improvement was observed when multi-breed imputation was performed.

**Conclusion:**

In all breeds, imputation accuracy was higher than 97%, which indicates that imputation to the high-density chip was accurate. Imputation accuracy depends mainly on the size of the reference population and the relationship between reference and target populations.

## Background

The development of a high throughput chip of 54 001 single nucleotide polymorphisms (SNPs) for cattle, the Bovine SNP50 BeadChip^®^ (50K), has drastically reduced genotyping costs and strongly contributed to the current implementation of genomic selection (GS). This approach, which was first proposed by Meuwissen et al. [1], uses a reference population (usually consisting of progeny-tested bulls) with both genotypes and phenotypes to estimate marker effects and then uses these estimates to predict breeding values for animals without phenotypes. The accuracy of prediction depends mainly on the size of the reference population, heritability of the phenotypes, and level of linkage disequilibrium (LD) between markers and quantitative trait loci (QTL) [2,3]. In some bovine breeds, a very large number of animals have been genotyped with the 50K chip and it is now possible to predict breeding values of animals at birth with high accuracy. In breeds with a limited number of progeny-tested bulls, assembling a large enough reference population is a real challenge. Under the assumption that LD is conserved across breeds, reference populations from different breeds can be combined to increase the size of the reference population. Conservation of LD, however, requires more than 300 000 informative SNPs and therefore, is not fulfilled with the classically used 50K chip [4]. The BovineHD BeadChip^®^ (HD) that was developed in 2010 and contains 777K SNPs is expected to be sufficiently dense to detect conserved LD across breeds and allow multi-breed GS. Re-genotyping all animals on this HD chip is, however, not economically feasible but prediction (imputation) of HD genotypes from 50K genotypes is possible.

Several imputation methods have been implemented and are widely used to impute genotypes from one chip to another. They are based either on population LD (Fastphase [5], Beagle [6], MaCH [7], IMPUTE2 [8]), or on a combination of LD and family information (Fimpute [9], Dagphase [10], AlphaImpute [11], FindHap [12]). Many studies have compared these methods for imputation from low-density panels to the 50K chip. Beagle 3.3.0 has been shown to be an accurate software package [13-15] and is commonly used in bovine datasets.

In this study, Beagle 3.3.0 was used to study the accuracy of imputation from the Illumina 50K to the HD chip in 16 French cattle breeds and to investigate the main factors that affect this accuracy.

## Methods

### Genotypes

The dataset comprised 5153 animals genotyped with the HD chip. The cryopreserved semen, or blood samples of the animals included in our study, which were used for genotyping, were procured from various commercial AI organizations and breeder organizations through their routine practice in the framework of breeding programs. Therefore, no ethical approval was required for sampling of biological material. The HD chip contains 777 964 markers with an average probe spacing of 3.45 kilobases (kb) [16]. Animals belonged to 16 breeds (seven dairy breeds and nine beef breeds). The number of genotypes was not equally distributed across breeds (Table 1) but depended on population size. Animals genotyped with the HD chip were chosen based on their marginal contribution to the population, as defined by Boichard et al. [17], and computed using the PEDIG software [18]. If possible, two to three progeny of each founder were also genotyped in order to increase phasing accuracy, and the most influential progeny were chosen for HD-genotyping. In some situations, and in particular in breeds with small populations, the number of HD-genotyped animals was constrained by the availability of DNA from ancestors. The family structure of each breed is detailed in Table 1. The mean number of HD-genotyped male progeny per bull was higher in dairy breeds (2.5) than in beef breeds (2). In the Holstein breed, a large proportion of genotypes originated from the Eurogenomics consortium [19,20]. Another dataset, containing 33 746 animals genotyped with the Bovine SNP50 BeadChip^®^[21], was also available. The genotypes originated either from the national genomic selection program or from complementary research programs, except for most of the Holstein genotypes, which were from the Eurogenomics consortium.

**Table 1:** Number of high-density genotyped animals and population structure per breed

	**Nb of genotyped animals**	**Nb of genotyped families (sire + progeny)**	**Mean nb of genotyped progeny per sire**	**Nb of effective ancestors**
**Dairy breeds**				
Abondance (ABO)	209	54	3.69	15
Brown Swiss (BSW)	99	52	1.90	28
Holstein (HOL)	788	204	2.30	21
Montbéliarde (MON)	530	139	3.77	18
Normande (HOR)	551	138	3.82	23
Simmental (SIM)	125	55	2.24	39
Tarentaise (TAR)	185	65	2.77	15
**Beef breeds**				
Aubrac (AUB)	254	116	2.17	112
Bazadaise (BAZ)	89	60	1.45	46
Blonde d'Aquitaine (BLA)	327	187	1.74	78
Charolais (CHA)	672	310	2.14	249
Gasconne (GAS)	163	76	2.12	197
Limousine (LIM)	462	235	1.96	185
Parthenaise (PAR)	304	97	3.02	89
Rouge des Prés (RDP)	149	80	1.83	99
Salers (SAL)	246	186	1.31	99

HD and 50k genotypes were used for parentage testing and to check the clustering quality of the HD genotypes by concordance analysis of the genotypes of the 1838 individuals genotyped on both chips.

### Data editing

Quality control was performed within-breed on the HD and 50K genotypes, using the same criteria for both chips. Genotyped animals with a call rate lower than 0.95 were removed from the analysis. Only markers mapped on the UMD3.1 assembly covering the 29 bovine autosomes were used for analysis. SNPs showing departure from Hardy-Weinberg equilibrium (p-value < 0.001) or with more than 10% missing genotypes were removed. In addition, genotype consistency was checked using 1838 animals that were genotyped on both chips and 352 markers that were discordant for more than 1% of these animals were excluded. After editing, 708 771 and 44 580 SNPs were retained on the HD and 50K sets, respectively. The 37 634 SNPs present on both HD and 50K sets were used to mimic 50K genotypes. Parentage was tested following the French routine genomic selection procedure, using both 50K and HD datasets (S. Fritz, personal communication). This procedure uses 500 informative markers and a parentage error was concluded if more than 10 incompatibilities were detected. In case of inconsistency, the progeny was removed, except when at least two progeny from the same sire were found incompatible with their sire. In this case, the sire was removed. Genotypes were checked for Mendelian inconsistencies between compatible parents and offspring. The genotype of the male parent was deleted if more than 20% of its progeny showed contradiction, in other cases the genotype of the progeny was set to missing.

### Assessing within-breed imputation accuracy

The accuracy of imputation from 50k to HD genotypes was computed within each breed. For this purpose, the HD-dataset was divided into two parts. The oldest animals were used as a training population to mimic a reference population genotyped with the HD chip. The 20% youngest animals formed the validation population. For animals in the validation population, markers that were only present on the HD chip were masked to mimic a target population genotyped with the 50K.

Beagle 3.3.0 was used to impute mimicked 50K genotypes up to high-density. This software uses a population-based method called “localized haplotype-cluster method”, as described by Browning and Browning [6]. The method builds and clusters haplotypes along the whole chromosome and then uses an underlying variable length Markov chain based on haplotypes counts (and consequently on local LD patterns) to determine transition probabilities from one marker to the next. The scale and shift parameters were set to 2 and 0.1, respectively, and no pedigree information was taken into account.

Imputation accuracy was estimated based on the comparison between imputed and known HD genotypes and was defined as the allelic imputation error rate, computed as the ratio between the number of falsely imputed alleles and the total number of imputed alleles [22].

Identification of SNPs with high error rates is important because they may induce errors in QTL detection and genomic selection. To identify these SNPs, only the breeds with the largest populations (Blonde d’Aquitaine, Charolais, Holstein, Limousine, Montbéliarde and Normande) were considered in order to avoid erroneous identification of errors due to the use of a low number of genotypes when computing the error rate for a SNP.

### Factors affecting imputation accuracy

Given the large number of breeds included here, it was possible to analyze the factors that may have an effect on imputation accuracy. Two types of variables that differ between breeds were studied: genetic diversity indicators such as the level of linkage disequilibrium and the number of effective ancestors, and variables specific to our datasets, such as the number of genotypes in the reference population and the relationship between reference and validation populations.

To avoid bias due to sampling, we used the indicators computed by Danchin-Burge [23] using the complete pedigree file of each breed. The number of effective ancestors was preferred to effective population size since the former is less sensitive to the depth of the pedigree [23].

Linkage disequilibrium was computed within-breed as a squared correlation coefficient based on phased genotypes for each marker pair [24], as defined in equation (1):

(1) $$r^{2}=\frac{{\left(p_{A1B1}-p_{A1}p_{B1}\right)}^{2}}{p_{A1}p_{A2}p_{B1}p_{B2}}$$

where A1, A2, B1, B2 are the alleles of SNP A and B, p_A1,_ p_A2_, p_B1_, and p_B2_ are the corresponding allelic frequencies, and p_A1B1_ is the frequency of the A1B1 haplotype.

For the Abondance breed, LD was computed for SNPs on *Bos taurus* (BTA) chromosomes 5, 10, 15, 20 and 25 and no differences between chromosomes were observed (data not shown). Therefore, for other breeds, LD computation was only performed on BTA5. Pairs of SNPs for which one SNP has a minor allele frequency (MAF) lower than 5% in the considered breed were discarded because it has been shown that LD tends to be very low in such cases [25]. Furthermore, removing SNPs with low MAF facilitates comparisons between populations [25,26]. Then, LD for all marker pairs were averaged by the distance between SNPs. We focused particularly on the level of LD between SNPs 70 kb apart, which corresponds to the average distance between two informative SNPs on the 50K chip.

Average relationship levels between training and validation populations were measured based on pedigree information using the PEDIG [18] software. A relationship coefficient was computed for each pair of individuals in each population and then averaged for each breed.

The effect of the different factors on imputation accuracy was assessed by multiple regression across all evaluated datasets (including both dairy and beef breeds). Tested factors were the reference population size, the number of effective ancestors, the linkage disequilibrium level at 70 kb and the relationship between training and validation populations. Factors were considered significant if the regression coefficient was different from zero (p-value < 0.05).

### Computation of multi-breed imputation accuracy

Since our aim was to improve imputation accuracy in breeds with a small reference population size, multi-breed imputation was tested. Training and validation populations from different breeds were combined and then imputation accuracy was computed as described above. Considering the diversity of the breeds involved in this project, it did not seem relevant to combine all breeds. Instead, breeds were grouped based on Reynolds genetic distances between breeds, which were computed by Gautier et al. [27] using allele frequencies on the 50K chip and represented in a Neighbor-Joining tree. One branch, which was composed of the Abondance, Montbéliarde and Tarentaise breeds, was taken as an example of closely related breeds and these breeds were merged for the multi-breed study. Imputation accuracy was then computed per animal and averaged for each breed.

## Results

### Linkage disequilibrium decay

**Figure 1:**
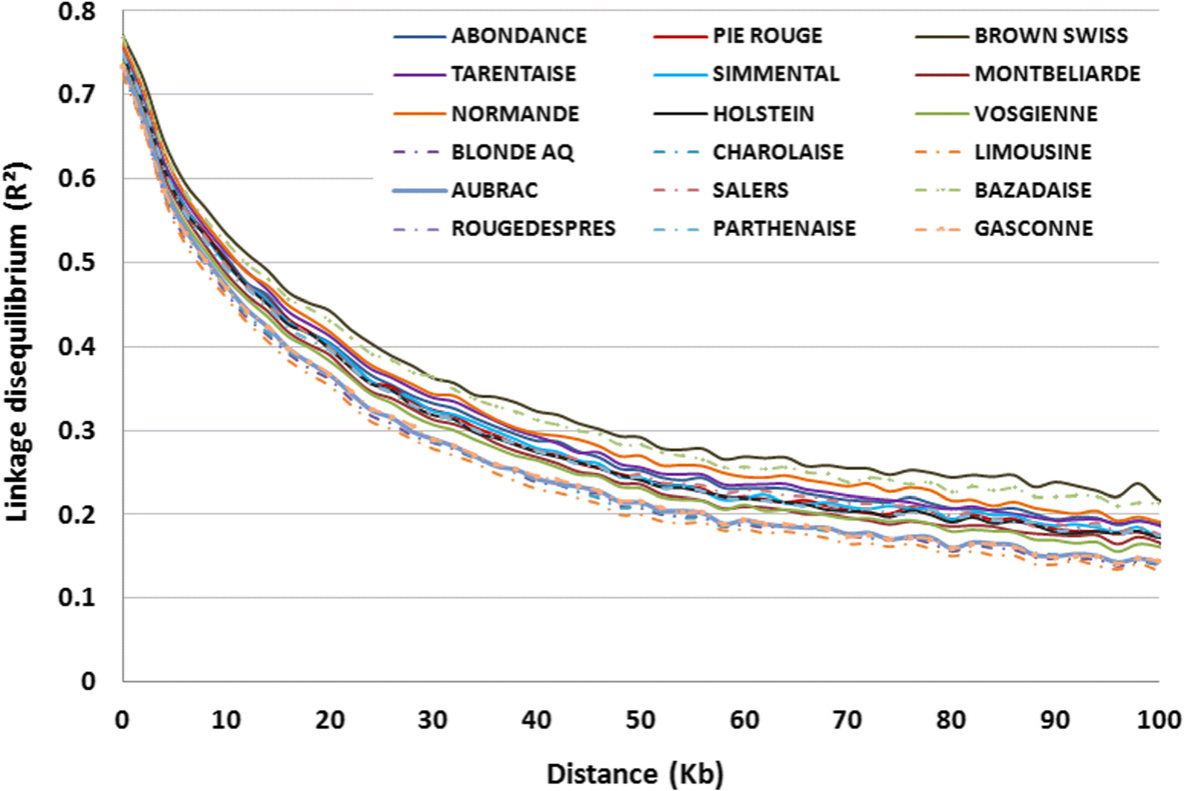
Linkage disequilibrium decay in 16 beef (dashed lines) and dairy (solid lines) cattle breeds.

The results of LD decay are presented in Figure 1. Within the first 10 kb, LD strongly decreased in all breeds, thereafter it decreased at a lower rate and differences between breeds became noticeable. LD levels at 70 kb (Table 2) varied from 0.16 in the Parthenaise breed to 0.26 in the Holstein breed; on average LD levels at 70 kb were higher by 0.04 in dairy breeds than in beef breeds, in agreement with the difference in number of effective ancestors between these breeds.

**Table 2:** Within-breed imputation error rate and others parameters affecting imputation error rate

	**Training population size**	**Validation population size**	**Allelic imputation error rate (%)**	**LD level at 70 kb**	**Average R**_**T/V**_
**Dairy breeds**					
Abondance (ABO)	169	40	0.75	0.217	0.146
Brown Swiss (BSW)	79	20	1.92	0.255	0.074
Holstein (HOL)	634	154	0.73	0.255	0.078
Montbéliarde (MON)	424	106	0.51	0.196	0.116
Normande (HOR)	444	107	0.33	0.233	0.104
Simmental (SIM)	100	25	2.55	0.209	0.050
**Beef breeds**					
Aubrac (AUB)	204	50	2.03	0.177	0.028
Bazadaise (BAZ)	72	17	2.07	0.239	0.038
Blonde d'Aquitaine (BLA)	262	65	1.80	0.175	0.038
Charolais (CHA)	539	133	0.68	0.176	0.018
Gasconne (GAS)	131	32	2.26	0.174	0.026
Limousine (LIM)	370	92	1.09	0.164	0.014
Parthenaise (PAR)	245	59	1.88	0.161	0.024
Rouge des Prés (RDP)	119	30	2.39	0.206	0.028
Salers (SAL)	197	49	1.27	0.213	0.024

Size of the training and validation populations, within-breed imputation error rate, level of linkage disequilibrium (LD, r^2^)) at 70 kb and average relationship between training and validation populations (R_T/V_).

### Imputation accuracy

Error rates were low in most breeds, with an overall mean error rate of 1.36% (Table 2). However, large differences were observed between breeds, with a minimum error rate of 0.31% in the Normande breed and a maximum of 2.41% in the Simmental breed. The error rates were less than 0.7% for breeds with more than 500 animals in the reference population.

Error rates were lower in dairy breeds (mean error rate = 1.02%) than in beef breeds (mean error rate = 1.62%). In beef breeds, the error rate ranged from 0.64% in the Charolais breed to 2.26% in the Rouge des Prés breed. In dairy breeds, error rates were higher in the Simmental and Brown Swiss breeds, which are regional breeds in France with many imported ancestors and limited numbers of genotyped animals.

### Factors affecting imputation accuracy

**Figure 2:**
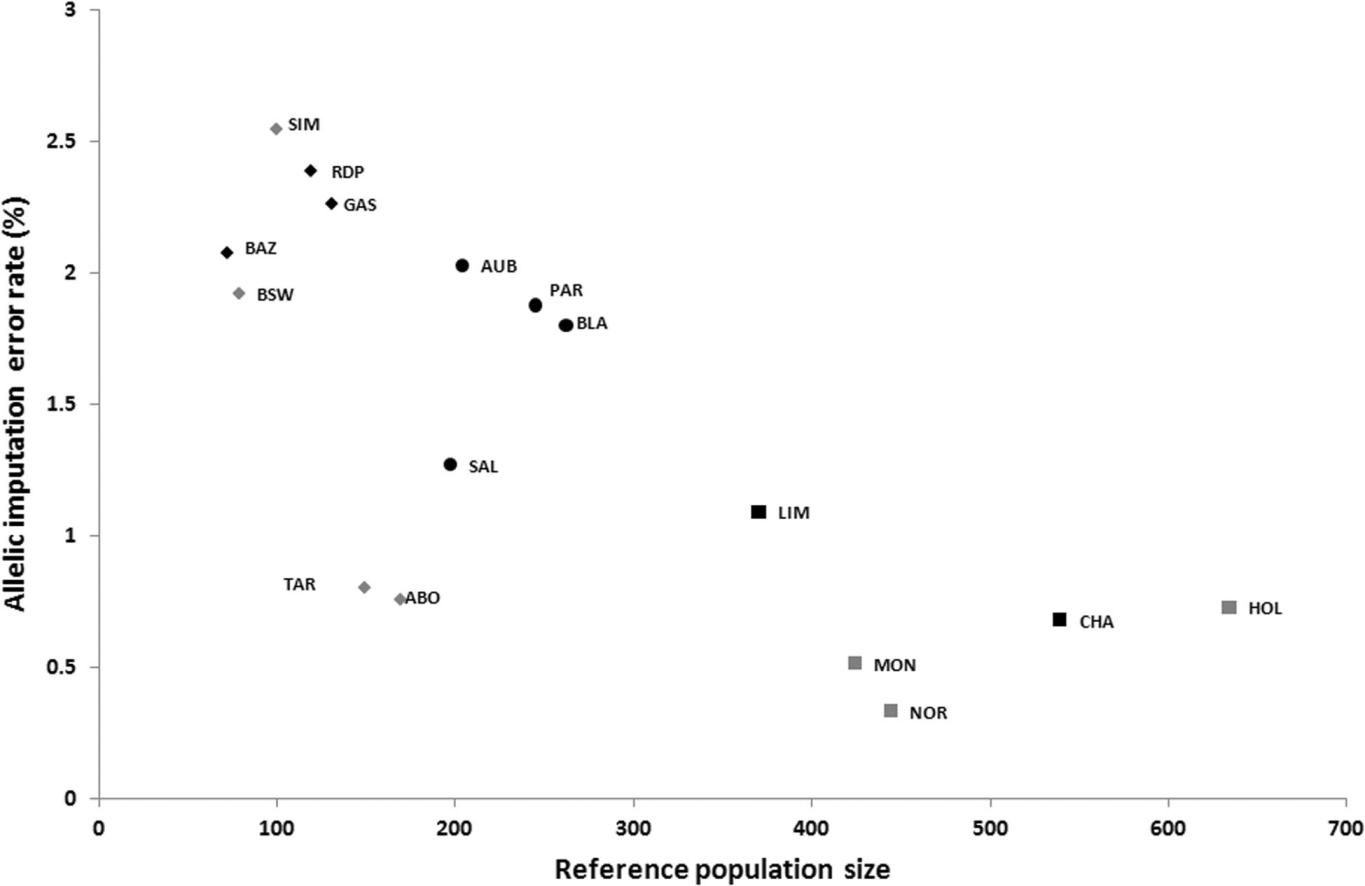
**Relationship between allelic imputation error rate and reference population size in beef (black) and dairy (gray) cattle breeds.** Breeds with more than 300 animals in the reference population are represented by a square, those with more than 200 animals by a circle, and those with 200 or less animals by a diamond.

Sizes of training and validation populations are presented in Table 2. Error rates decreased linearly when the number of animals in the reference population increased (Figure 2). Again, we observed a higher accuracy in dairy breeds: an error rate lower than 1% was achieved with only 100 to 200 genotyped animals in dairy breeds, whereas approximately 400 animals were required for beef breeds. With more than 400 genotyped animals, the impact of reference population size on accuracy leveled off, which suggests that the effect of size of the reference population is non-linear when a critical size is reached.

**Figure 3:**
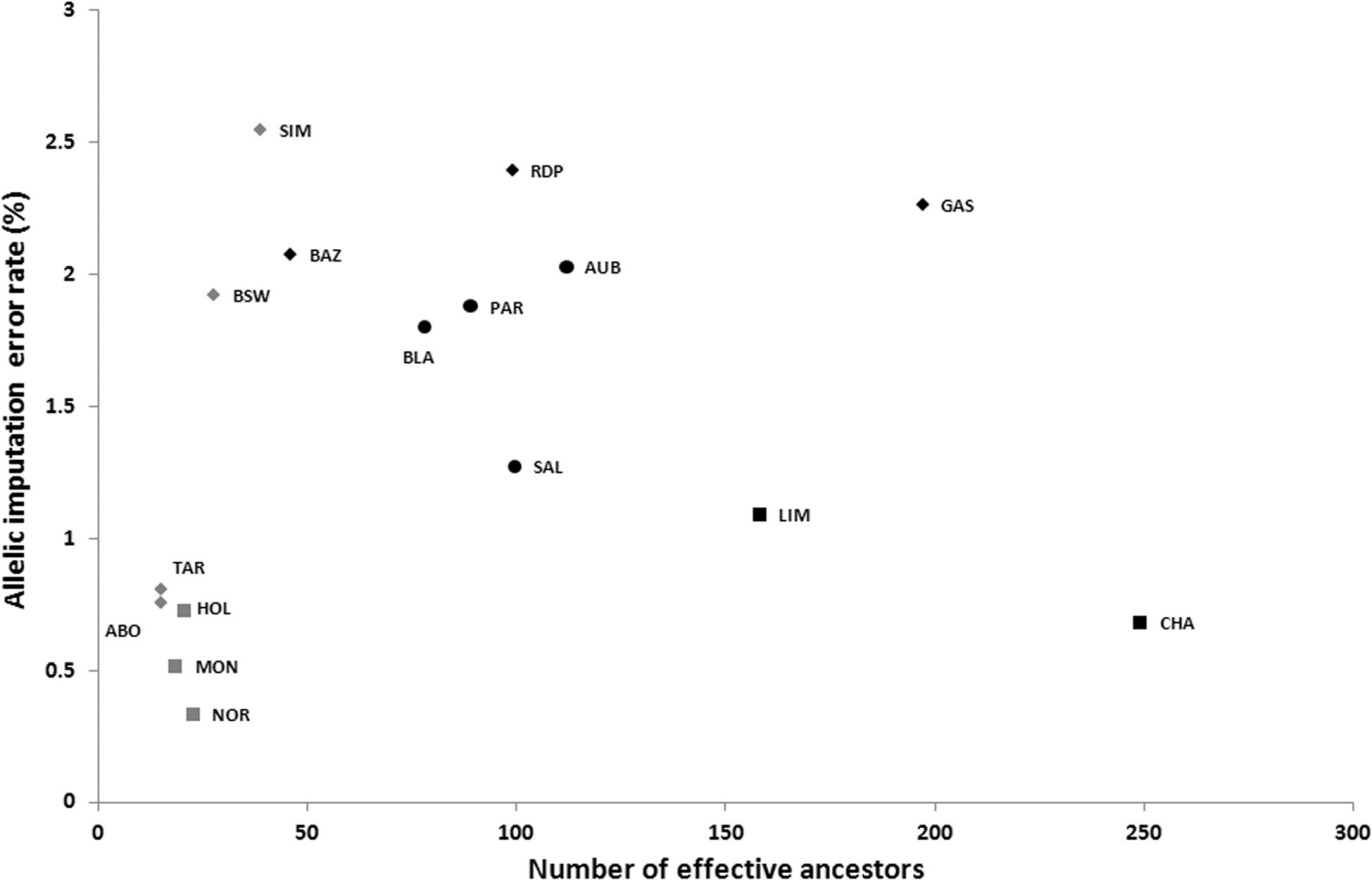
**Relationship between allelic imputation error rate and number of effective ancestors in beef (black) and dairy (gray) cattle breeds.** Breeds with more than 300 animals in the reference population are represented by a square, those with more than 200 animals by a circle, and those with 200 or less animals by a diamond.

The relationship between the number of effective ancestors and imputation error rate is illustrated in Figure 3. The number of effective ancestors appeared to have an opposite effect on dairy versus beef breeds. In dairy breeds, the number of effective ancestors was low (< 50) and its relationship with imputation error rate was positive. However, the two breeds with the highest number of effective ancestors (Simmental and Brown Swiss) among the dairy breeds had the highest imputation error rate but also the smallest reference populations and many foreign ancestors were not genotyped. In beef breeds, a negative relationship between the number of effective ancestors and imputation error rate was observed, but the breeds with the highest number of effective ancestors (Charolais and Limousine) also had the largest reference populations. This suggests that the effect of the number of effective ancestors was masked by the effect of the reference population size. However, when considering populations with equal reference population sizes (e.g. Limousine and Montbeliarde), the error rates increased with number of effective ancestors.

**Figure 4:**
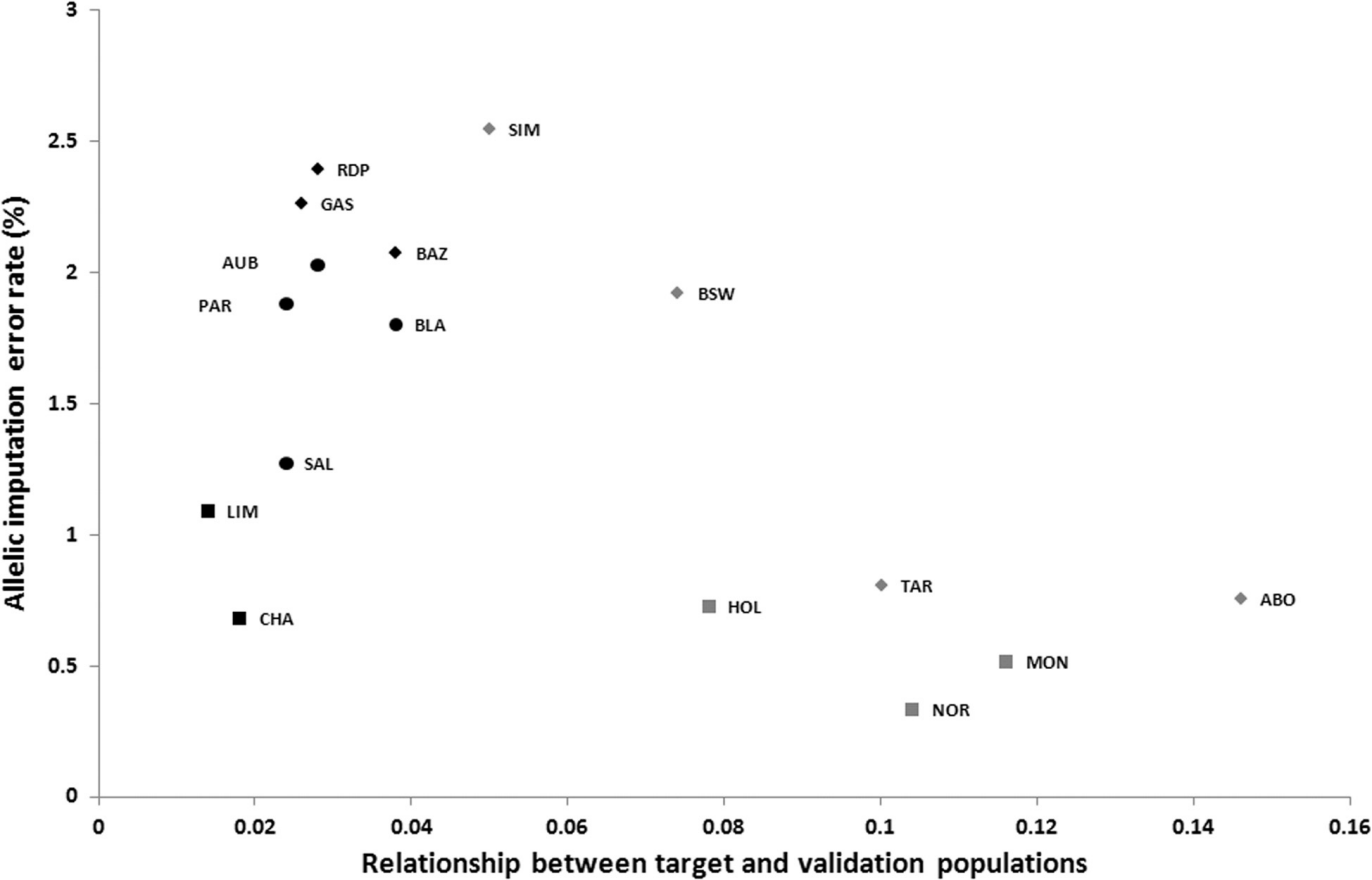
**Relationship between allelic imputation error rate and the genetic relationship between target and validation populations in beef (black) and dairy (gray) cattle breeds.** Breeds with more than 300 animals in the reference population are represented by a square, those with more than 200 animals by a circle, and those with 200 or less animals by a diamond.

Figure 4 presents the relationship of the imputation error rate with the degree of genetic relationship between training and validation populations (R_T/V_). The first observation is that R_T/V_ was clearly lower in beef than in dairy breeds, which may be explained by a higher number of effective ancestors in beef breeds. The second observation was that when breeds with the largest populations were discarded, the relationship between R_T/V_ and imputation error rate appeared to be linear. Among breeds with large populations, the relationship between training and validation populations had only a limited effect on the imputation error rate. The Charolais and Limousine breeds had low imputation error rates despite their lower R_T/V_ but these breeds have large reference populations. However, the lower R_T/V_ in the Holstein breed probably explains why the error rate was higher in this breed than in the Normande or Montbeliarde breeds.

Finally, multiple linear regression was performed to better quantify the impact of each factor on imputation accuracy. Results of the multiple linear regressions are in Table 3. Reference population size and R_T/V_ had a significant effect (p < 0.05) on imputation error rate, in contrast to the number of effective ancestors and the level of LD. About half of the variation in imputation error rates was explained by reference population size and one quarter by R_T/V_. Using the regression coefficients, we can predict that increasing R_T/V_ by 1% reduces imputation error rate by 0.12% and that adding 100 animals to the reference population decreases the error rate by 0.26% (Table 3). This suggests that the size of the reference population is the major factor affecting imputation error rate but the relationship between training and validation populations must also be taken into consideration. Despite the difference between breeds, our results suggest that the number of effective ancestors and the level of LD are not major factors affecting imputation accuracy.

**Table 3:** Results of the multiple linear regression model

	**Part of variance explained**	**Regression coefficient**	**p-value**
Reference population size	52%	−0.0026 ± 0.0006	0.002
Relationship between reference and validation populations	25%	−12.6042 ± 3.8937	0.008
Level of linkage disequilibrium at 70 kb	2.5%	−0.0028 ± 0.0026	0.305
Number of effective ancestors	0.03%	−0.0576 ± 4.4641	0.899

The model tests the effect of reference population size, relationship between reference and validation populations, level of linkage disequilibrium at 70 kb, and number of effective ancestors on imputation error rate.

### SNP by SNP analysis of imputation accuracy

**Figure 5:**
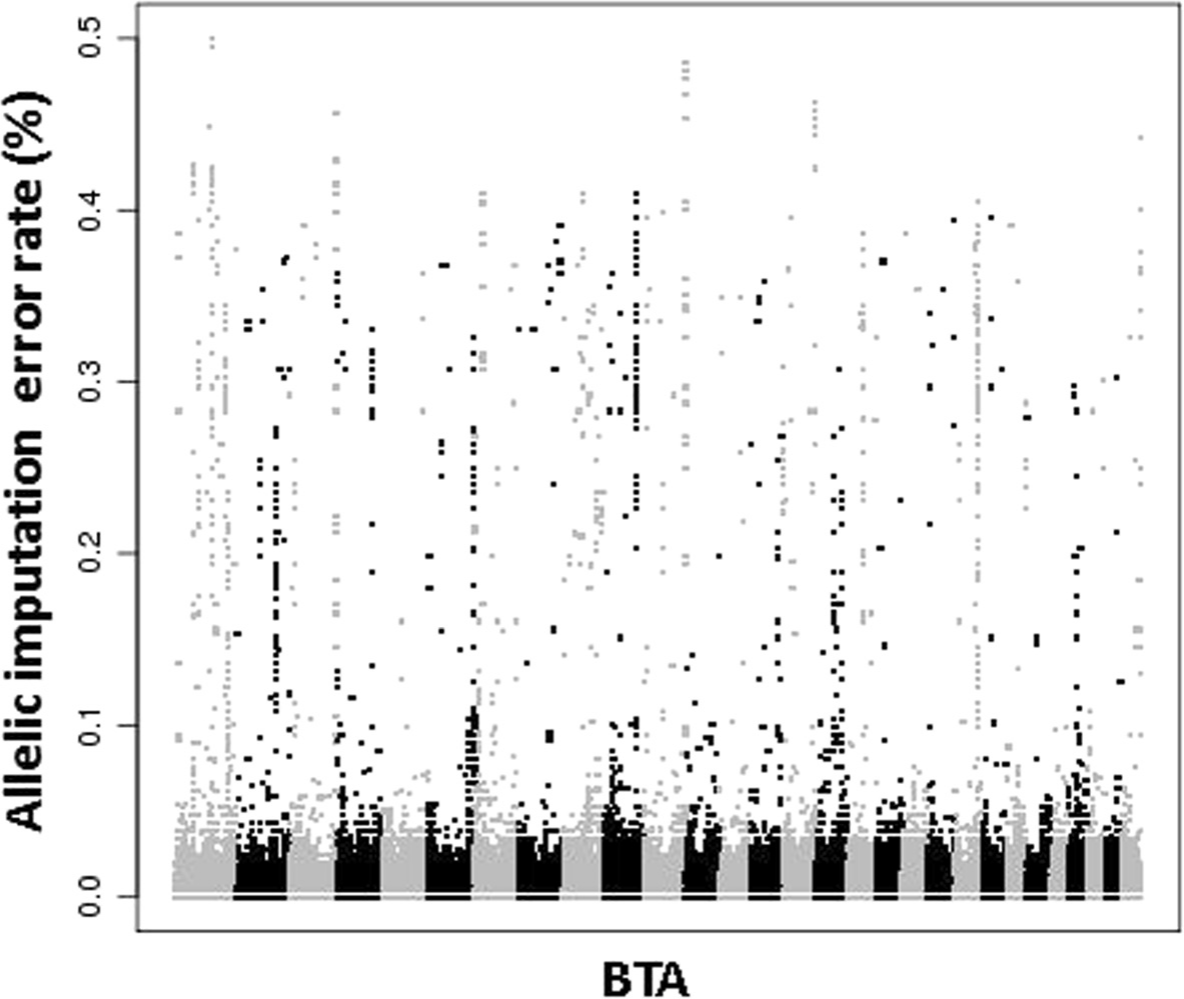
Allelic imputation error rate along the genome in Montbéliarde breed.

Imputation error rate was computed for each SNP in order to detect SNPs that were mismapped in the UMD3.1 assembly. This analysis was performed for the six major breeds (three beef breeds and three dairy breeds). Results in the Montbéliarde breed are presented in Figure 5. Despite an overall mean error rate of 0.51%, 13 104 and 6030 SNPs had error rates greater than 5 and 20%, respectively. Consequently, the error rate dropped from 0.51 to 0.40% after removing potentially mismapped SNPs with error rates greater than 5%.

**Figure 6:**
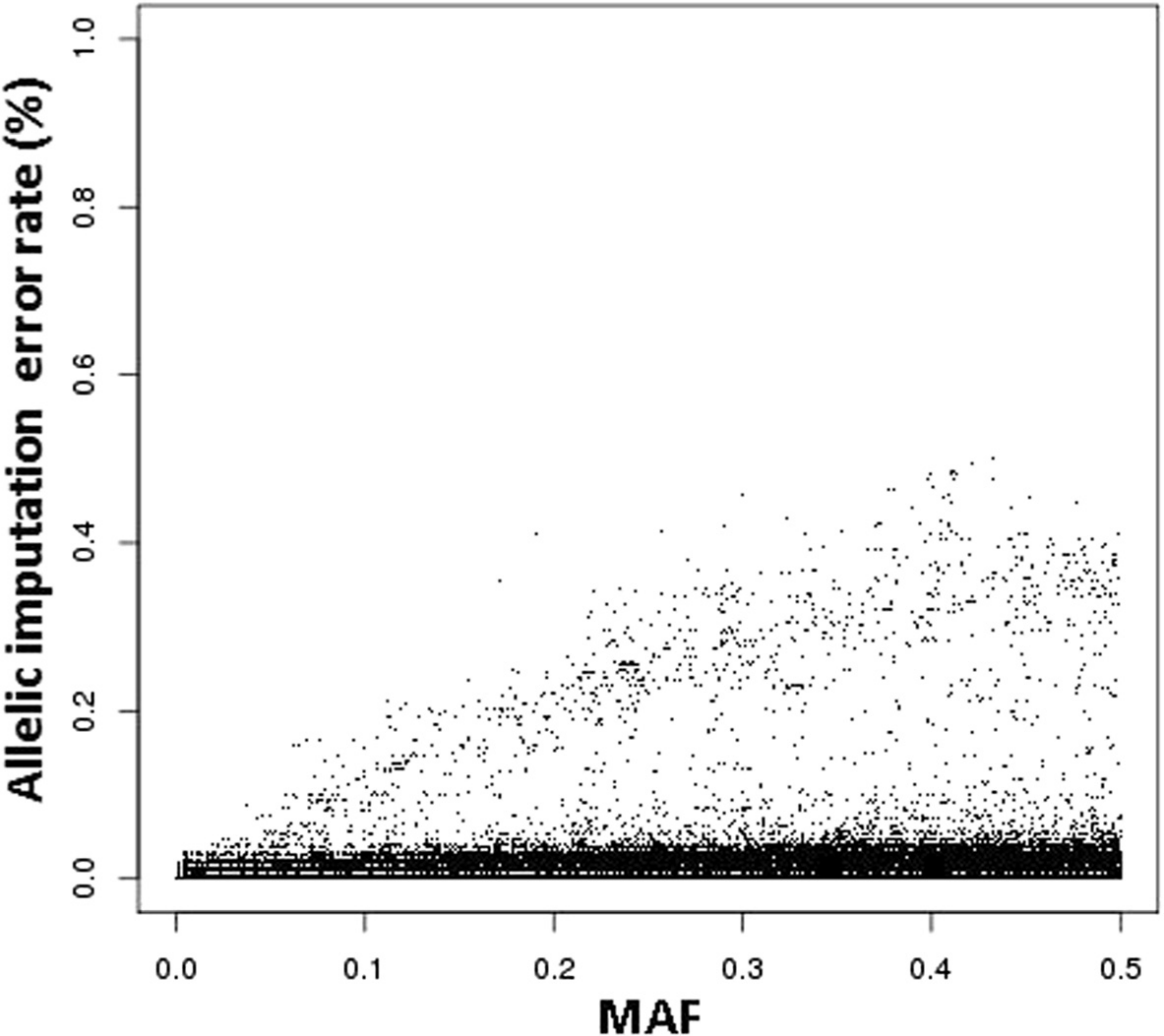
Relationship between allelic imputation error rate and minor allele frequency in Montbéliarde breed.

The relationship between MAF and imputation error rate is illustrated in Figure 6 for the Montbéliarde breed. SNPs were divided in two groups based on their imputation error rates. No relationship with MAF was detected for SNPs with error rates less than 0.1, whereas error rates increased with MAF for SNPs with high error rates. Assuming that the LD between a mismapped SNP and its direct neighbors is low, a mismapped SNP is imputed by chance and therefore its error rate is related to its MAF.

We looked for SNPs with high error rates in each breed based on a threshold that was defined as the mean error rate plus 3 times its standard deviation for that breed. Three thousand and eighty-three, 1980, and 1146 SNPs had high error rates in at least two, three or six breeds, respectively. SNPs with high error rates in at least three breeds are likely mismapped and are listed in Additional file 1: Table S1.

### Multi-breed imputation accuracy

Since imputation accuracy depends highly on the number of HD genotypes, we increased the size of the reference population by combining breeds. The results for single- and multi-breed situations are in Table 4. No difference in accuracy was found between single- and multi-breed imputation for the Abondance and Montbéliarde breeds. For the Tarentaise breed, imputation error rate was slightly higher in the multi-breed analysis than in the single-breed analysis.

**Table 4:** Imputation error rate using single-breed populations compared to a multi-breed reference population for three breeds

	**Abondance**	**Montbéliarde**	**Tarentaise**
Size of training / validation population	159 / 40	422 / 106	146 / 36
Single-breed imputation error rate (%)	0.755	0.487	0.763
Multi-breed imputation error rate (%)	0.753	0.485	0.824

## Discussion

In this study, we evaluated accuracy of imputation from 50K to HD-genotypes for 16 cattle breeds and we investigated the corresponding causes of variation. We observed large differences in imputation accuracy between breeds. Several factors may explain these differences, i.e. size of the reference population and the relationship between training and validation populations (closely related to population structure). Results from the multiple linear regression performed in this study, combined with other published results, lead us to propose several hypotheses on the impact of each factor.

The number of HD genotypes in the reference population ranged from 72 in the Bazadaise breed to 634 in the Holstein breed and is the major factor that explains differences in imputation accuracy between breeds. The imputation error rate decreased by 0.26% when 100 animals were added to the reference population. However, other studies have shown a non-linear effect of reference population size on imputation error rate. In Holstein cattle, Schrooten et al. (unpublished data) reported that imputation error rate decreased by between 0.17 and 0.04% when moving from 200 to 500 HD genotypes by steps of 100, while in sheep, the decrease was 5% when moving from 50 to 150 individuals in the reference population but only 2% when moving from 204 to 2512 individuals [28]. In our study, the effect of the size of the reference population on imputation error rate was linear. This effect would probably have been found non-linear if more breeds with a large reference population had been included. In fact, the size of the reference population had a limited effect when the number of HD genotypes was greater than a minimum threshold that was estimated at 200–400 animals.

Hayes et al. [28] reported that most of the differences in imputation accuracy are due to differences in the relationship between the reference and target populations and the genetic diversity of the breed. Schrooten et al. (unpublished data) used traceability, defined as “the expected contribution of HD-genotyped ancestors to the genotype of an animal”, as a measure of the relationship between the reference population and one animal from the target population. Imputation error rates were lower for animals with higher traceability, meaning that a higher average traceability, i.e. a higher relationship between the reference and target populations, will result in lower error rates. We reached the same conclusion, i.e. the imputation error rate was decreased by 0.12% when the average relationship increased by 0.01.

Increasing the size of the reference population decreases the probability to miss a haplotype in the reference population. For a fixed reference population size, an increase in the number of effective ancestors, i.e. an increase in the number of haplotypes in the total population, increases the probability to miss a haplotype and thus increases the error rate. This explains why reference population size and number of effective ancestors had opposite effects on imputation accuracy and compensate for each other. In our study, differences in within-breed diversity explain why the error rate was higher in beef breeds than in dairy breeds and why more than 99% accuracy was achieved with only 200 animals for dairy breeds, while 400 animals were necessary for beef breeds. The poor results obtained with the Simmental and Brown Swiss breeds were also due to lower relationships between the reference and target populations; in these breeds, key ancestors mainly originate from abroad and were not included in our data. Combining reference populations from different breeds did not improve imputation accuracy, which confirms the results on multi-breed imputation of Hayes et al. [28] and Erbe et al. [29]. In fact, multi-breed imputation is expected to improve imputation accuracy only when 50K haplotypes are conserved across breeds, which is quite unlikely given the history of the breeds and their estimated divergence time, even for the most closely related breeds.

Although observed imputation error rates were low in all breeds, 1980 SNPs had particularly high error rates (Figure 5 and Additional file 1: Table S1), which suggests that errors exist in the marker map. Erbe et al. [29] identified 1231 SNPs with genotype error rates greater than 20%. When some of these SNPs were remapped using LD, error rates dropped. In our study, no remapping was performed but removing SNPs with high error rates resulted in a 0.1% drop in error rate, which suggests that lower error rates can be achieved with a more accurate map. However, Erbe et al. [29] still found 630 poorly imputed SNPs after remapping, which means that other reasons, such as recombination hot spots or regions on the 50K panel with lower SNP density, explain the high imputation error rates for some SNPs. SNPs with high imputation error rates likely also impact the quality of genomic selection and QTL detection. This has not been specifically investigated, but some studies have focused on the impact of imputation from low-density panels on reliability of genomic selection [14,30] and concluded that an imputation error rate between 2 and 3% leads to a mean loss of reliability of 2%. For imputation from 50K to the HD chip, the mean error rate is close to 1%, which suggests that the impact on reliability of genomic selection is even lower. However, because of the large number of markers available after imputation, it is preferable to discard markers with high error rates.

## Conclusions

The mean error rate for imputation from the Illumina Bovine50K^®^ to the BovineHD^®^ was around 1%. Differences in error rates between breeds were large and ranged from 2.41% in the Simmental breed to 0.31% in the Normande breed. These differences were mainly due to the size of the reference population and the relationship between the reference and target populations. This means that imputation accuracy could be increased by increasing the number of HD genotypes and by improving the reference population to maximize its relationship with the population to impute. Using 50K genotypes to impute HD genotypes is possible, which implies that a large HD imputed reference population can be available for genomic selection at low cost. However, new HD genotypes are likely required in the future generations in order to maintain relationship links between the reference and target populations and limit the imputation error rate.

## Competing interests

The authors declare that they have no competing interests.

## Authors’ contributions

CH and MNF performed the analysis. DB, VD, FP and PC designed the study. EV and SF managed the databases. FG and RD provided computer programmes. CH, DB, VD, PC wrote the manuscript. All authors read and approved the final manuscript.

## Supplementary Material

Additional file 1 **High_Error_Rate_SNP.** Csv file with header and separator ‘;’ containing the list of markers with high imputation error rate. Columns are as follows: chromosome number, SNP name, number of breeds for which a high error rate was detected and average error rates across breeds. Click here for file
